# Revealing Hidden Length by Force: Decoupling Modulus and Toughness in Network Gels

**DOI:** 10.1021/acscentsci.5c01718

**Published:** 2025-09-23

**Authors:** Shakkeeb Thazhathethil, Xiaoran Hu

**Affiliations:** Department of Chemistry, BioInspired Institute, Syracuse University, Syracuse, New York 13244, United States

## Abstract

Reactive strand extension decouples toughness from modulus in single- and double-network gels.

## Graphical abstract

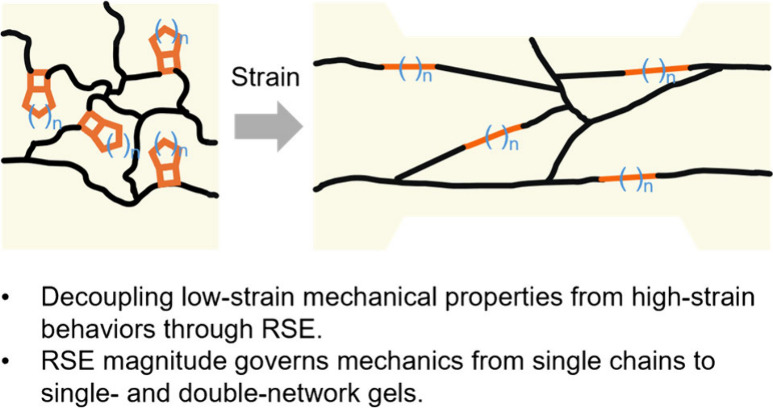

Cross-linked polymer networks underpin everything from tires, seals, and adhesives to contact lenses and soft robotic actuators, where performance hinges on surviving application-specific load histories. A primary determinant of the lifetime and usefulness of polymer network materials—including elastomers and hydrogels—is their ability to resist fracture initiation and growth under repeated mechanical deformation. When strain exceeds a critical threshold, flaws nucleate cracks that subsequently propagate to catastrophic failure. At the molecular level, crack propagation is resisted by a small population of highly stretched polymer strands at the crack tip; propagation requires the energy to stretch these individual polymer strands to the point at which they break, typically via homolytic bond scission.

## Hidden Length, Revealed Impact

Building on this molecular picture, chemists from the NSF Center for the Chemistry of Molecularly Optimized Networks have introduced reactive strand extension (RSE),^1,2^ an elegant network-design strategy that could one day transform brittle polymer networks into stretchable, resilient materials without compromising stiffness. In their earlier work,^1^ Wang et al. reported polymer double networks (DNs) incorporating bicyclic cyclobutane mechanophore units within the constituent strands that undergo force-coupled [2 + 2]­cycloelimination to release stored “hidden” length. This mechanochemical reaction allows polymer strands to lengthen when overstretched, preserving covalent connectivity and avoiding (or delaying) force-induced chain scission at their nominal breaking point. As a result, RSE enables more strands to engage synergistically at the crack tip to resist fracture. By the time an RSE strand ultimately breaks, it will have absorbed and dissipated more energy through force-coupled cycloeliminations than an analogous strand lacking RSE capability.

## Hierarchical Experimental Methodology: From Single Molecules to Networks

In this issue of *ACS Central Science*,^3^ Gong, Sottos, Craig, and co-workers present a comprehensive, hierarchical study that advances fundamental understanding of how force-coupled covalent RSE effects in polymer strands translate into the macroscopic properties of network polymers. They first synthesize random copolymers P5 and P12 (the numerals denote the number of atoms in the fused ring) by radical copolymerization^4^ of the corresponding bicyclic cyclobutane monomers with NaAMPS and an acetoacetate methacrylate handle to promote robust AFM attachment in single-molecule force spectroscopy (SMFS). Molecular models estimate ∼5.9 Å of added contour length per cycloelimination for P5 and ∼15.2 Å for P12. Stretching single chains at ∼1.5–2.0 nN reveals reproducible plateaus where stored length is released, amounting to ∼44% of the initial contour length for P5 and ∼85% for P12—close to, but still below, the theoretical maxima (58% and 150%, respectively).

In this issue of ACS Central Science, Gong, Sottos, Craig, and co-workers present a comprehensive, hierarchical study that advances fundamental understanding of how force-coupled covalent RSE effects in polymer strands translate into the macroscopic properties of network polymers.

Remarkably, the same chemistry observed in single-molecule experiments is translated into network materials with predictable behaviors. The authors report the first clear observation of the RSE effect in single-network (SN) gels, improving their stretchability and toughness: keeping low-strain properties comparable, the network built from strands with the larger RSE “step size” reaches the highest ultimate strain. In DN hydrogels, where a prestretched primary network carries the early load before transferring stress to a softer secondary network, the same ranking holds: more hidden length in the first network delays breakage and pushes the material farther.

## Highlights

As a design concept, RSE is theoretically important and practically powerful because it decouples low-strain mechanical properties from high-strain behaviors. Most toughening strategies either soften the material (sacrificial ionic bonds, unfolding domains) or complicate formulation (fillers, phase separation). By contrast, RSE offers a rare escape from this dilemma: little happens until strands approach their contour limit, then force activates the chemistry that unveils the hidden length in the RSE strands instead of breaking the polymer strands, preserving network connectivity while redistributing stress over a larger number of resisting strands. This strategy improves the toughness of materials at the nominal breaking point without affecting their low-strain modulus, making RSE an empowering tool for designing next-generation network materials whose modulus and toughness (which are typically anticorrelated) can be tuned independently and predictably.

As a design concept, RSE is theoretically important and practically powerful because it decouples low-strain mechanical properties from high-strain behaviors.

Setting this study apart is not just the promise of RSE as a design strategy for tougher gels but also the systematic, hierarchical methodology that tracks the RSE effect from the single molecule into SN organogels and finally into DNs. This work provides a quantitative molecule-to-material structure–property blueprint: rather than invoking a generic “RSE effect,” we can now specify the ångströms of hidden length released per mechanochemical event, the magnitude of forces that gate it, and how these parameters affect and correlate with bulk properties such as tearing energy and ultimate strain. This work demonstrates that precise control of strand-level reactivity can have tangible, predictable, and tunable consequences at the network level.

**1:**
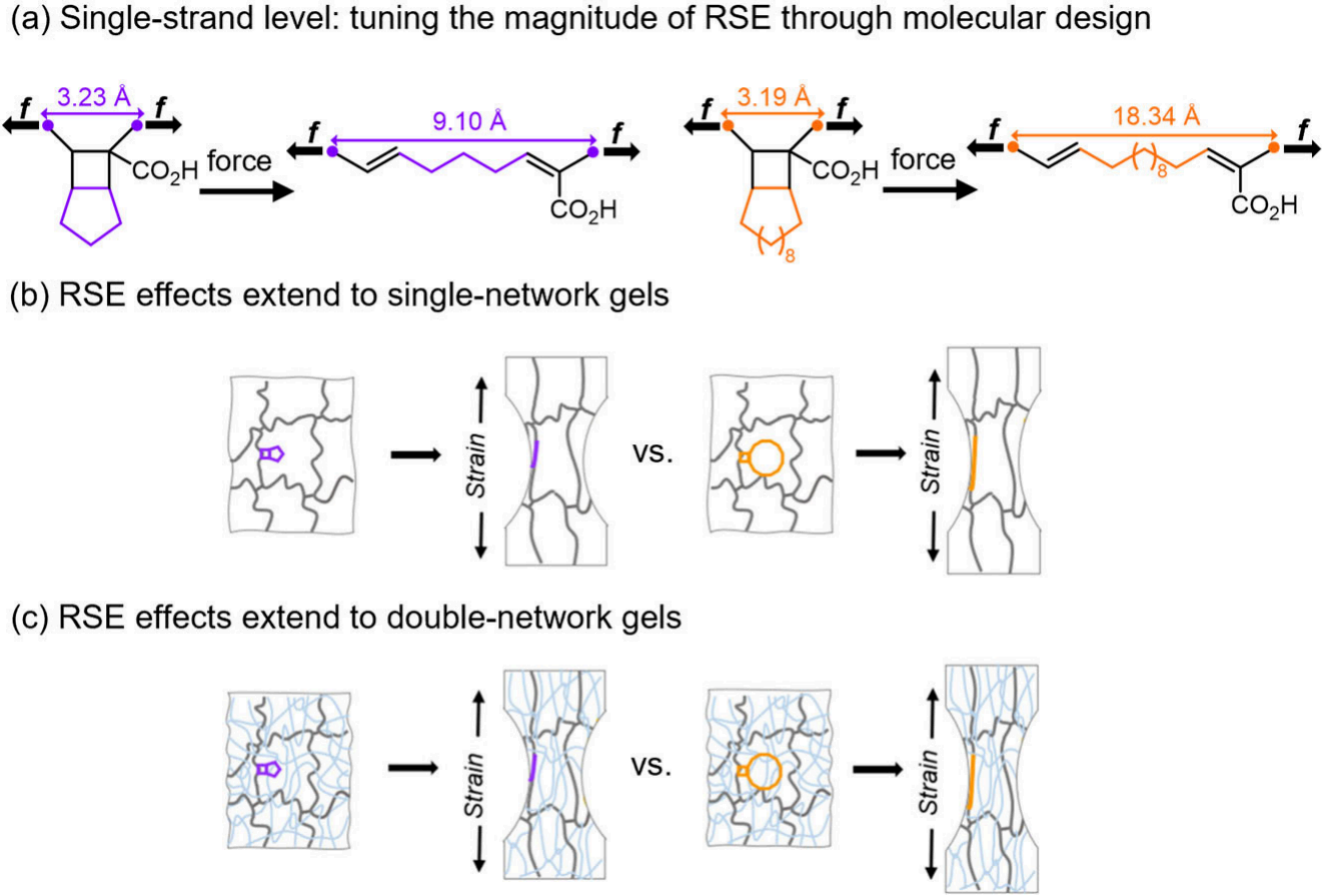
Schematic of reactive strand extension (RSE) and its cross-scale impact: single chains (a), single-network (b), and double-network (c) gels. Adapted with permission from ref 3. Available under a CC-BY 4.0 license. Copyright 2025 Xujun Zheng, Chun-Yu Chiou, Sunay Dilara Ekim, Tatiana B. Kouznetsova, Jafer Vakil, Yixin Hu, Liel Sapir et al.

Setting this study apart is not just the promise of RSE as a design strategy for tougher gels, but also the systematic, hierarchical methodology that tracks the RSE effect from the single molecule into SN organogels and finally into DNs.
